# Opportunities and Challenges in the Development of Cathode Materials for Rechargeable Mg Batteries

**DOI:** 10.3389/fchem.2018.00634

**Published:** 2018-12-18

**Authors:** Jan Bitenc, Robert Dominko

**Affiliations:** ^1^Department of Materials Chemistry, National Institute of Chemistry, Ljubljana, Slovenia; ^2^Faculty of Chemistry and Chemical Technology, University of Ljubljana, Ljubljana, Slovenia; ^3^ALISTORE - European Research Institute, Cedex, France

**Keywords:** batteries, magnesium, cathode, sulfur, organic redox compounds

## Abstract

Recent years have seen an intense and renewed interest in the Mg battery research, naming Mg-S the ≫Holy Grail≪ battery, and expectations that Mg battery system will be able to compete and surpass Li-ion batteries in a matter of years. Considerable progress has been achieved in the field of Mg electrolytes, where several new electrolytes with improved electrochemical performance and favorable chemical properties (non-corrosive, non-nucleophilic) were synthesized. Development in the field of cathodes remains a bit more elusive, with inorganic, sulfur, and organic cathodes all showing their upsides and downsides. This review highlights the recent progress in the field of Mg battery cathodes, paying a special attention to the performance and comparison of the different types of the cathodes. It also aims to define advantages and key challenges in the development of each type of cathodes and finally specific questions that should be addressed in the future research.

## Introduction

Electrical energy storage (EES) is a key technology of the imminent future, and one of the necessary requirements for the broader application of renewable energy resources. There is a plethora of EES technologies like different battery systems, fuel cells, supercapacitors, electrolyzers. However, the most mature and likely dominant EES technology in coming decades will be the Li-ion battery. Concerns about the availability of Li and other metals used in contemporary Li-ion batteries are inciting researchers to develop other technologies that would enable use of batteries on all application levels ranging from electromobility, stationary energy storage to robotics and of course portable electronics.

Magnesium is a promising material for the future post Li-ion batteries due to the high gravimetric (2,206 mAh/g) and volumetric capacity (3,834 mAh/cm^3^) of Mg anode. Non-dendritic deposition of Mg enables use of Mg metal anode in the practical Mg batteries. The higher redox potential of Mg, when compared with Li, decreases the overall voltage of the battery, but this is compensated by the high capacity of the anode, given the condition that capacity of the cathode does not decrease. Moreover, Mg is among ten most abundant elements in the Earth's crust, which should remove any concerns about sustainability of Mg batteries. Besides discussed benefits, there are several challenges connected with the use of Mg metal, mainly incompatibility of the Mg metal with the electrolytes commonly used in Li-ion batteries and lack of suitable cathodes. Mg deposition from solutions of Grignard reagent was reported already in early twentieth century (Gaddum and French, 1927). Unfortunately, the low conductivity and inadequate oxidative stability prevents their application as battery electrolytes. In 1990, Gregory et al. reported on the use of various Mg complexes and inorganic compounds that could function as electrolytes and cathodes, respectively (Gregory et al., 1990). A proof-of-concept Mg battery was demonstrated by Aurbach et al. who utilized a Mo_6_S_8_-Chevrel phase as a cathode and Mg(AlCl_2_BuEt)_2_ as an electrolyte (Aurbach et al., 2000). The battery displayed impressive long-term cycling, it could operate for more than 2,000 cycles with a capacity fade of < 15%. The voltage of this system was relatively low displaying two plateaus at 1.3 and 1.1 V. This seminal work of Aurbach et al. inspired others, and demonstrated the need to develop cathode materials with higher voltages. Along with development of new electrolytes, a lot of effort has been devoted into exploration of different magnesium insertion compounds based on oxide, polyanionic, or chalcogenide structures, with only limited success. On the other hand, the recent discoveries of non-nucleophilic electrolytes (Kim et al., 2011; Zhao-Karger et al., 2013, 2017; Doe et al., 2014) opened a path toward the use of electrophilic cathode materials like S and organic compounds, which are quickly catching up and in some characteristics also outperforming inorganic cathodes.

## General Properties of Mg Anode

**Figure 1:**
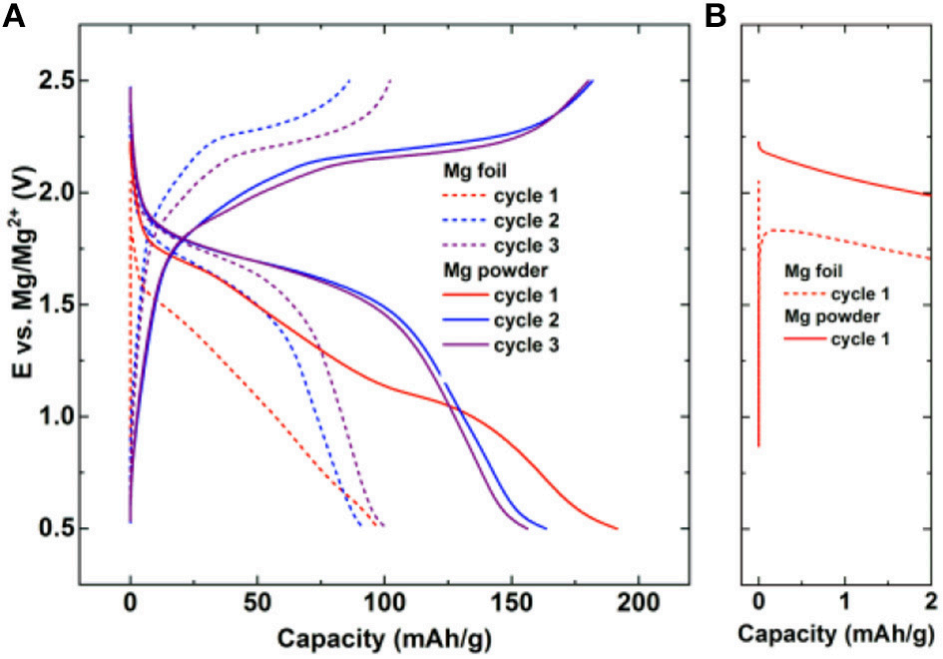
Galvanostatic cycling of Mg-organic battery with poly (anthraquinoyl sulfide) cathode and two different Mg anodes: non-brushed Mg foil (dashed) and synthesized Mg powder (full line). **(A)** Selected galvanostatic cycles at a current density of 50 mA/g in Mg_2_Cl_3_-HMDSAlCl_3_ in tetrahydrofuran (THF). **(B)** Inset into the first discharge at the start of the cycle. Reproduced with permission from Bitenc et al. (2015). Copyright Wiley-VCH.

Due to thermodynamic instability of Mg and Li metals in electrolytes, a passive film formation is typically formed at the interphase. However, unlike passive film on Li which is good ionic conductor, Mg passive film typically tends to block the transport of the Mg^2+^ ions, which narrows the choice of salts and solvents for Mg electrolytes. Impurities in small amounts like H_2_O lead to the formation of compact passive films on Mg anode, which can significantly increase the overpotentials for Mg stripping/deposition (Connell et al., 2016; He et al., 2017). Thus, attention should be paid to minimization of water content in Mg electrolytes by using extra dry salts and solvents. Partially passivating Mg electrolytes [Mg(TFSI)_2_ without any additives] lead to large overpotentials for Mg stripping/deposition (Ha et al., 2014; Tutusaus et al., 2017). Practical cycling of 2-electrode Mg cells containing Mg anodes is impossible in electrolytes containing carbonates, nitriles, and Mg(ClO_4_)_2_ due to complete passivation of the Mg metal. Besides electrolyte components, the morphology and pretreatment procedures of Mg anode also affect the stripping/deposition process at the anode. This is illustrated in Figure 1 where the electrochemical behavior of Mg-organic battery is shown for two different types of anode. The cell with non-brushed Mg (unmodified native passive film) foil exhibits a higher overpotential in the initial cycles compared to the cell with synthesized Mg powder anode (Figure 1A—difference between doted and full line curves). The difference in the overpotential is particularly high at the start of the first cycle (Figure 1B) and lowers with continued cycling. Type of the Mg anode is especially important in chloride free electrolytes, which have limited capability for Mg surface etching.

## Inorganic

Inorganic materials considered for Mg cathodes span from chalcogenides, oxides to polyanionic structures. While inorganic materials have already been reviewed in considerable detail (Canepa et al., 2017), we wish only to highlight the most important developments that represent advancement in understanding of Mg intercalation inside well-organized inorganic framework. From the early reports by Aurbach et al. on Mg intercalation into Mo_6_S_8_ (Aurbach et al., 2000), this material has served as a benchmarking material and is by far the most studied Mg cathode. Facile extraction of Mg ions from Mg_2_Mo_6_S_8_ at room temperature is possible only for the first Mg whereas it is more or less impossible for the second one due to trapping of Mg ions inside the structure (Levi et al., 2006). This effect can be mitigated by replacing S with Se, which allows a full capacity utilization of Mo_6_Se_8_ (Levi et al., 2005).

**Figure 2:**
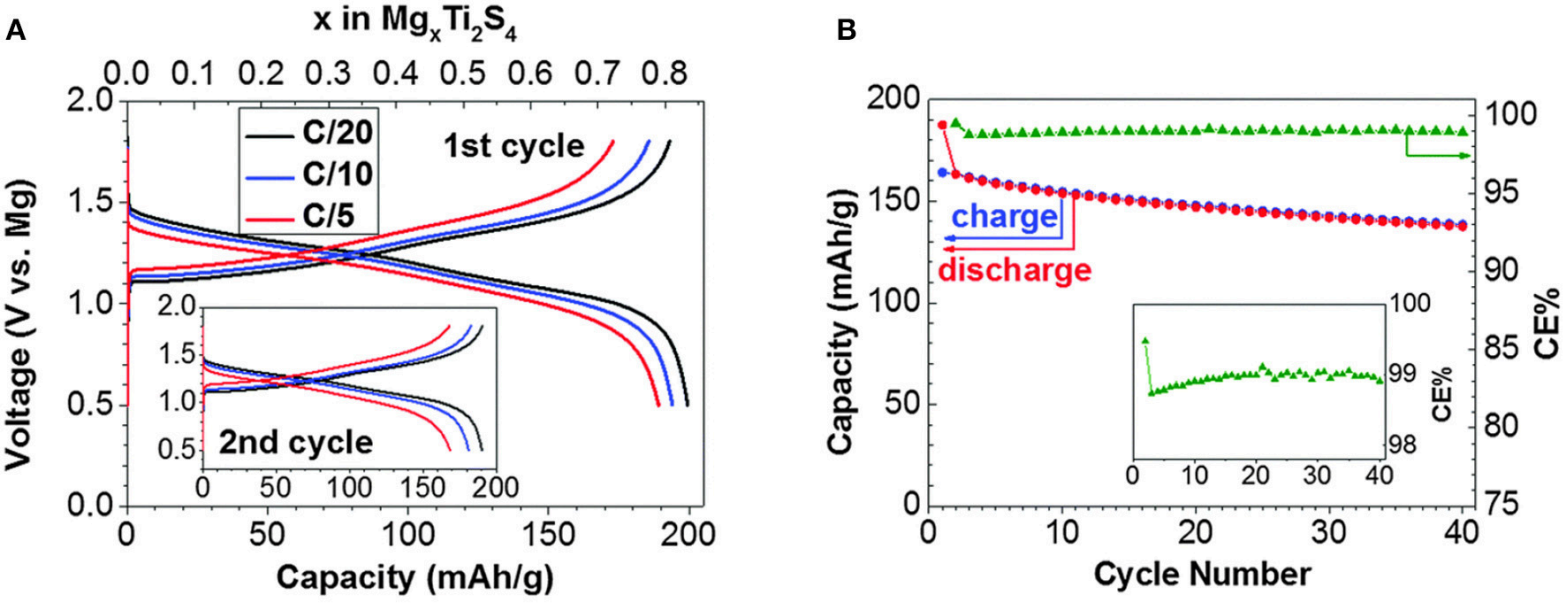
**(A)** First and second (inset) galvanostatic cycles of thiospinel Ti_2_S_4_ in all phenyl complex (APC) in THF electrolyte at 60°C. **(B)** Capacity and Coulombic efficiency of Ti_2_S_4_ at a 0.1C rate in APC in tetraglyme (TEG) electrolyte at 60°C. Reproduced with permission from Sun et al. (2016a). Copyright Royal Society of Chemistry.

Other chalcogenides offer an opportunity of surpassing the electrochemical performance of Chevrel phases by using other transition metals, while maintaining low electrostatic interactions between the Mg ion and the anion lattice (Wang et al., 2015). Low electrostatic interactions lead to increased Mg ion mobility, but this comes at a cost of reduced cell voltage. One of the most studied materials are titanium sulfides which can be divided into three groups: bulk layered (TiS_2_), spinel (Ti_2_S_4_), or nanotubes (TiS_2_). Early results exemplified pronounced hysteresis (Amir et al., 2007) or showed questionable 2-electrode cycling in passivating Mg electrolytes (Tao et al., 2004). Recent reports have demonstrated that both thiospinel Ti_2_S_4_ (Sun et al., 2016a) and layered TiS_2_ (Sun et al., 2016b) can display good reversibility at elevated temperature of 60°C. Especially thiospinel, with its relatively good cycling stability represents considerable improvement over both capacity and voltage of the Chevrel phase cathode (Figure 2). The second most researched chalcogenide material is MoS_2_ (Li and Li, 2004; Liang et al., 2011, 2015). Among selenides TiSe_2_ (Gu et al., 2015) and WSe_2_ (Liu et al., 2013) have been examined. However, the latter was tested in an electrochemical window that exceeded the operating window of the electrolyte.

Use of oxide materials opens a possibility to increase both the working voltage and capacity of the cathode materials. In practice most of the oxide materials suffer from poor Mg mobility (Rong et al., 2015; Sai Gautam et al., 2015), oxide conversion reactions (Zhang et al., 2015), pseudocapacitance and lack of suitable electrolytes that would enable reliable testing of their electrochemical activity. Certain layered materials like MoO_3_ (Spahr et al., 1995; Gershinsky et al., 2013), Mo_2.48_VO_9.93_ (Kaveevivitchai and Jacobson, 2016), and V_2_O_5_ (Gershinsky et al., 2013) have displayed reversible intercalation in electrolytes that cannot enable reversible Mg deposition/stripping. Solvent co-intercalation or intercalation of Mg^2+^ in hosts containing water have shown a beneficial effect on electrochemical performance for different MnO_2_ polymorphs [birnessite (Nam et al., 2015; Sun et al., 2016c) and spinel (Kim et al., 2015)] and V_2_O_5_ materials (Novak et al., 1999; Imamura and Miyayama, 2003; Tepavcevic et al., 2015).

Materials based on polyanionic structure are a very diverse group whose electrochemical performance is far from being fully explored. Early reports have shown a promising performance of different silicates (Mn, Fe, Co) (Feng et al., 2008; Li et al., 2011; NuLi et al., 2011). Although corrosion of Cu current collectors is the likely cause for identical redox potentials of silicates containing different transition metals and extremely large discrepancy between calculated and measured cathode potentials (Ling et al., 2012). Cycling of MgFeSiO_4_ prepared through the electrochemical delithiation of Li_2_FeSiO_4_ has demonstrated limited Mg insertion (Orikasa et al., 2014), which confirms contributions from side reactions in earlier reports. NASICON types of materials have been investigated with limited success (Makino et al., 2001; Huang et al., 2015) and have demonstrated magnesiation only on the surface of the particles (Huang et al., 2015). Intercalation of Mg into FePO_4_ caused amorphization of FePO_4_ due to thermodynamic instability of the intercalated product (Zhang and Ling, 2016). Among fluoro-polyanions, tavorite-FeSO_4_F should theoretically have good Mg mobility (Wu et al., 2014), while experimental results on MgFePO_4_F display limited electrochemical performance (Huang et al., 2014). An interesting group of materials are Prussian blue and its analogs that demonstrated the ability to intercalate several different multivalent ions both in aqueous and non-aqueous electrolytes (Gao et al., 2015; Lipson et al., 2016; Chen et al., 2017).

At the present state Chevrel phases and some other transition metal sulfides possess good reversibility of magnesium insertion while materials based on oxide and polyanionic structure cannot offer electrochemical performance required for practical cathodes due to poor mobility and structural instability. However, they might offer a prospect of developing high voltage Mg cathodes. Additional aspect, which has to be taken into consideration are interfaces between electrolyte and active cathode particles, particularly in chloride-containing electrolytes that are still used in many different experimental set-ups. A significant computational effort has been invested in the computer driven screening of the candidate inorganic hosts and could offer useful guidelines for future development (Emly and Van der Ven, 2015; Gautam et al., 2015; Rong et al., 2015; Sai Gautam et al., 2016; Kulish et al., 2017). Still, key requirements like good Mg^2+^ mobility inside intercalation host, long-term cycling stability and electrolyte compatibility will have to be demonstrated experimentally.

## Organic Materials

Organic materials are an attractive group of cathode materials, because they can be produced from low-cost and abundant resources, which in turn opens a path to more sustainable EES (Schon et al., 2016). The fact that they can be used with a variety of counter ions makes them especially interesting for the Mg system. Their downside is low volumetric energy density, which is compensated by high volumetric energy density of Mg anode. A rapid capacity fade due to dissolution of organic molecules into electrolyte remains a general issue. However, it can be effectively mitigated by grafting of organics onto a solid support (Genorio et al., 2010), preparation of insoluble polymers (Song et al., 2009), or use of selective separators (Senoh et al., 2011).

Organic materials often contain electrophilic centers inside their structures, which makes them incompatible with nucleophilic Mg electrolytes. However, there have been reports, where nucleophilic electrolytes are used with organic cathodes, but it is difficult to differentiate between electrochemical activity attributed to organic compounds and pseudocapacitance response from side reactions in this class of electrolytes (NuLi et al., 2007; Qiang et al., 2013). The first reversible electrochemical reaction of an organic compound with Mg^2+^ ions was demonstrated on dimethoxybenzoquinone (DMBQ) in 0.5 M Mg(ClO_4_)_2_ in γ-butyrolactone and confirmed through *ex-situ* XRD (Sano et al., 2012). Electrolyte used in this study passivates the Mg anode and reversible cycling of the cathode was achieved in a 3-electrode setup. Attempts of cycling organics in Mg passivating electrolytes in 2-electrode setups led to huge overpotentials for Mg stripping/deposition, which resulted in extremely poor cycling (Ha et al., 2014; Senoh et al., 2014, 2015).

**Figure 3:**
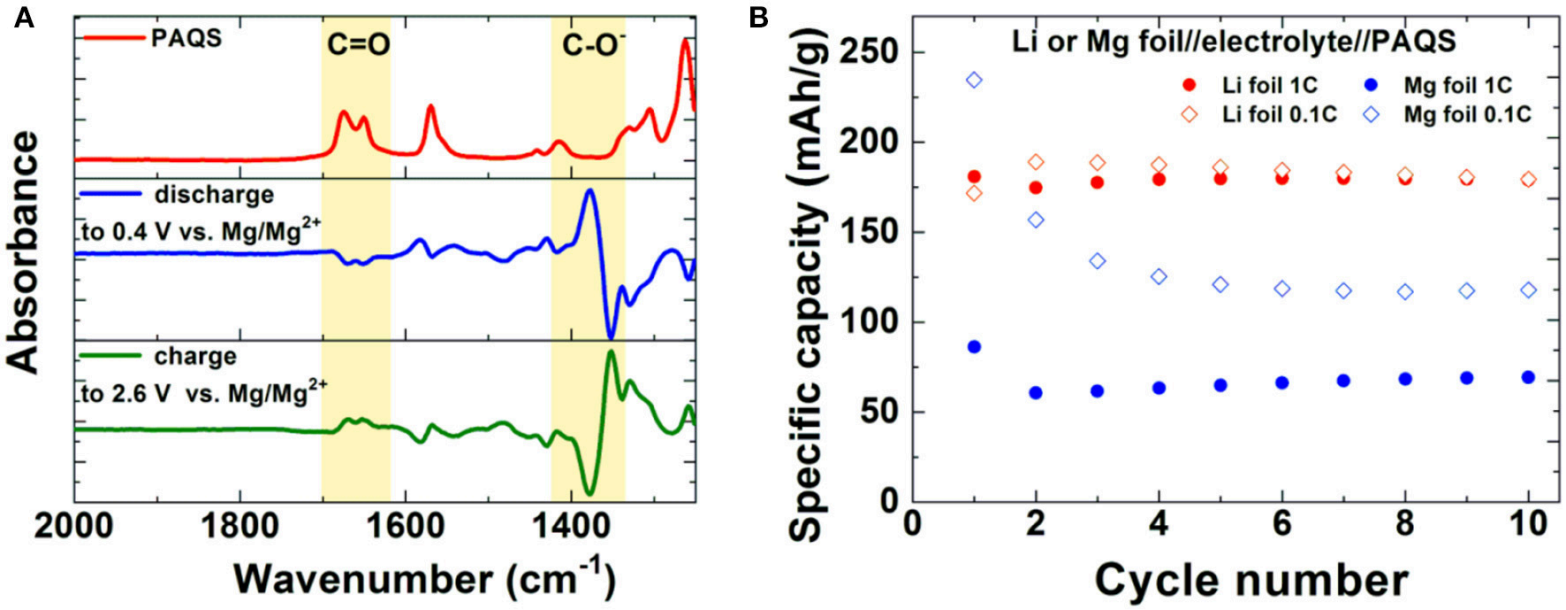
**(A)** ATR-IR spectroscopy of PAQS (red), *operando* ATR-IR of PAQS cathode in discharge (blue) and charge (green). **(B)** Capacity performance of PAQS cathode in Li and Mg battery system at different cycling rates in 0.4 M MgCl_2_-Mg(TFSI)_2_ in tetraglyme:dioxolane (1:1 vol%) (Vizintin et al., 2018). (Adapted from (Vizintin et al., 2018) under a Creative Commons attribution 4.0 International license. http://creativecommons.org/licenses/by/4.0/).

A significant step forward was achieved by a combination of non-nucleophilic electrolytes and an organic polymer cathode—poly(anthraquinonyl sulfide)-PAQS (Bitenc et al., 2015). This combination allowed Mg stripping/deposition with reasonable overpotential and improved capacity retention of the cathode compared to previous results. Average discharge voltage of the cell was 1.5 V. The electrochemical mechanism of Mg-PAQS system was investigated through *operando* ATR-IR (attenuated total reflectance infrared spectroscopy) which confirmed the reduction of carbonyl bond upon discharge (Vizintin et al., 2018). This is evident from the difference ATR-IR spectra of PAQS cathode obtained by subtraction of two subsequent spectra at different state-of-charge (Figure 3A). A decrease of intensity for C = O band and appearance of new –C–O^−^ band is observed during discharge, while in charge the intensity of C = O band increases and –C–O^−^ band decreases. This observation confirms a reversible electrochemical mechanism through reduction of carbonyl bond. At a current density corresponding to 1C (Figure 3B) significantly lower capacities were obtained in Mg system compared to the Li counterpart by using same type of cathode composite, which points toward worse accessibility of electroactive groups in the former. Differences between obtained capacities in Li and Mg batteries are lower at lower current densities, but still remain relatively large for this polymer.

The use of non-nucleophilic electrolytes has been extended to DMBQ which in combination with Mg exhibited, for the first time, a discharge voltage of 2.0 V. However, dissolution of DMBQ, which is a small, relatively easily soluble molecule, into electrolyte caused a rapid capacity fade (Pan et al., 2016b). Due to high voltage and high specific capacity of the benzoquinone group, polymers based on benzoquinone could be considered promising cathode materials. Indeed, our study involving such polymer has demonstrated an average discharge voltage of 2.0 V as well as a good cycling stability (Bitenc et al., 2016). Here it has to be noted that for benzoquinone based polymers practical capacities close to theoretical ones (around 400 mAh/g) have still to be achieved not only for Mg, but also for Li systems.

**Figure 4:**
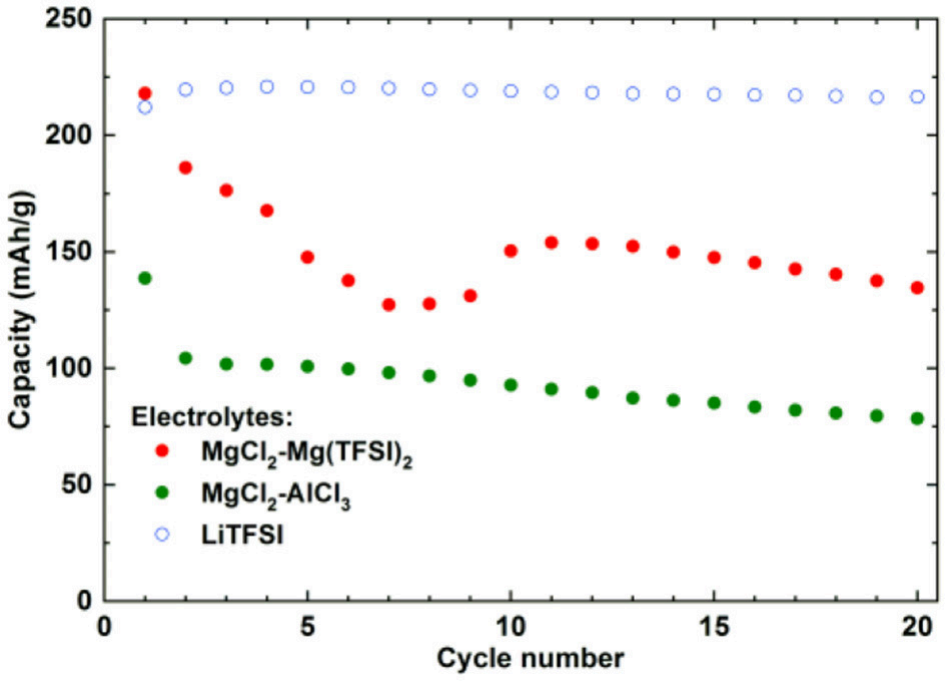
Comparison of capacity retention of PAQS in Li 1 M LiTFSI in DME:DOL = 1:1 (vol%) and two Mg electrolytes [Mg(TFSI)_2_-2.5MgCl_2_, MgCl_2_-0.3AlCl_3_] at current density of 50 mA/g in voltage window from 0.5 to 2.5 V. Adapted with permission from Bitenc et al. (2015). Copyright Wiley-VCH.

Impressive results have been obtained using 1,4-polyanthraquinone (PAQ), which showed superior cycling properties, i.e., more than 1,000 cycles at elevated rates (1C and 2C) in an electrolyte containing Mg(HMDS)_2_-4MgCl_2_ (HMDS-hexamethyldisilazane) and THF (Pan et al., 2016a). Obtained capacity in this work represents only 50% of the theoretical value (260 mAh/g) and the authors did not report on capacities that could be obtained in a comparable Li system (Song et al., 2015), which could serve as a reference point providing additional information about the practical utilization of this material. Our results on PAQS show that there are significant differences between the capacities that can be obtained in different Mg electrolytes and a standard Li electrolyte (Figure 4).

All the Mg-based electrolytes displayed lower capacities than the Li reference electrolyte. However, the differences among the Mg electrolytes were very high, the Mg(TFSI)_2_ based electrolyte has a maximum capacity of 218 mAh/g, while maximum capacity in AlCl_3_ containing electrolyte is significantly lower, only 139 mAh/g. Although capacity fade is faster in Mg(TFSI)_2_ this electrolyte still has 50% bigger capacity after 20 cycles. Maximum attainable capacity is also relatively close to the theoretical capacity (225 mAh/g) of PAQS. All this facts illustrate that while the Mg(TFSI)_2_ salt can partially passivate the Mg anode (Tutusaus et al., 2017), it is probably the optimal salt for organic cathode at the present state of electrolyte development since the maximum capacity in Mg system can be achieved using a Mg(TFSI)_2_ based electrolyte. These results demonstrate that the active species in Mg electrolytes depend on the used salts and have a significant effect on the practical performance of organic cathodes. However, significant steps forward will be needed in the direction of improving the capacity retention. Recently, we investigated polyimide cathode materials, where we successfully demonstrated electrochemical activity of naphthalene-hydrazine diimide polymer. The polymer undergoes reversible reduction to enolate anion, which was confirmed using *operando* ATR-IR. This exemplifies that Mg-organic batteries are not limited to only quinone class of compounds, but can be easily applied to other type of compounds (Bančič et al., 2018). Polyimide based material has also been used as an anode in aqueos Mg battery, which utilized Prussian blue cathode (Chen et al., 2017). In aqueous Mg electrolyte polyimide cathode could be cycled for more than 2,000 cycles.

Organic electrode materials offer good electrochemical performance in practical 2-electrode cells, which is summarized in Table 1. Currently, some of them exhibit long-term cycling performance, while others offer a possibility to achieve 2 V Mg batteries by application of the benzoquinone group (Bitenc et al., 2016). The next steps should focus on improving the capacity retention and achieving practical capacities close to theoretical ones. Results discussed above indicate that in certain combinations of active materials and electrolytes this goal is realistic. A practical realization of Mg-organic battery will also depend on the volumetric density, areal loadings of organic electrodes and the amount of electrolyte required for practical cells. Thus, future research efforts on the topic of organic materials should also address these questions.

**Table 1:** Summary of different organic compounds tested in 2-electrode setups with their theoretical capacity, average discharge voltages, capacity retention, and used electrolytes.

**Structure**	**Theor. capacity (mAh/g)**	**Average discharge voltage (V)**	**Maximum practical capacity (mAh/g)**	**Capacity retention (mAh/g) [number of cycles]**	Electrolyte and solvent^*^	**References**
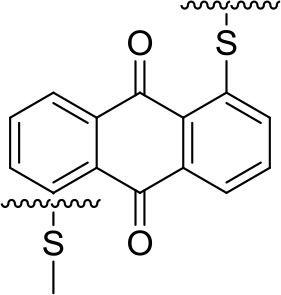	225	1.5 V	218	54 [100]	Mg(TFSI)_2_-2.5MgCl_2_ THF:DME = 2:3vol%	Bitenc et al., 2015
		1.5 V	139	42 [100]	MgCl_2_-0.3AlCl_3_ THF	Bitenc et al., 2015
		1.5 V	100	30 [100]	Mg(HMDS)_2_-4MgCl_2_ THF	Pan et al., 2016a
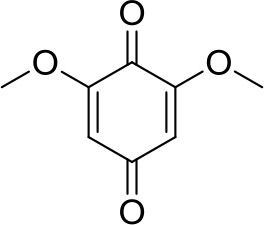	319	2.0 V	226	74 [30]	Mg(TFSI)_2_-2MgCl_2_ DME	Pan et al., 2016b
		0.45 V	100	50 [10]	Mg(TFSI)_2_ DEG	Pan et al., 2016b
		2.0 V	200	80 [5]	Mg(TFSI)_2_ DME, worse performance for higher glymes	Senoh et al., 2015
		below 0.5 V	100	20 [50]	Mg(TFSI)_2_ sulfolane	Senoh et al., 2014
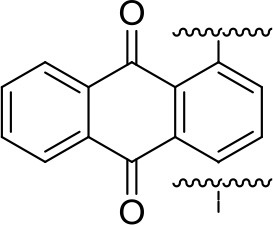	260	1.5 V	133	106 [100]	Mg(HMDS)_2_-4MgCl_2_ THF	Pan et al., 2016a
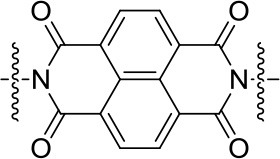	203	1.7 V	85	85 [100]	Mg(TFSI)_2_-MgCl_2_ TEG:DOL = 1:1 vol%	Bančič et al., 2018
		1.7 V	86	65 [100]	Mg(TFSI)_2_-MgCl_2_ TEG	Bančič et al., 2018
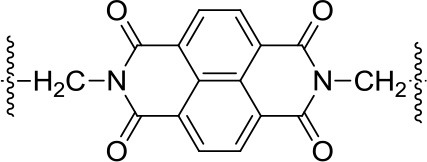	183	−0.5 V vs. Ag/AgCl	140	126 [2,000]	MgSO_4_ H_2_O	Chen et al., 2017
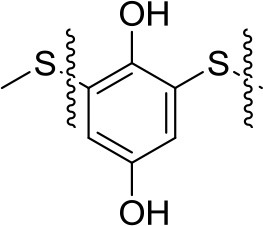	382	2.0 V	158	143 [25]	Mg(TFSI)_2_-1.5MgCl_2_ TEG:DOL = 1:1 vol%	Bitenc et al., 2016
		1.9 V	123	116 [40]	Mg(TFSI)_2_-1.5MgCl_2_ TEG	Bitenc et al., 2016

* *Solvent abbreviations: THF, tetrahydrofuran; DME, dimethoxyethane; DEG, diglyme; TEG, tetraglyme; DOL, dioxolane*.

## Sulfur

Sulfur is a promising cathode material for several battery systems such as Li-S, Na-S, and Mg-S. It offers a high gravimetric capacity of 1,672 mAh/g, which is ~10 times higher than the capacities of conventional insertion cathodes. It is considered as a cheap and sustainable cathode material. Its downsides are a lower voltage and a relatively low volumetric energy density. Thus, the Mg anode with its high volumetric capacity is an ideal anode to be coupled with the sulfur cathode. First Mg-S battery was reported jointly with first non-nucleophilic Mg electrolyte, Mg_2_Cl_3_-HMDSAlCl_3_ (Kim et al., 2011) with this electrolyte Mg-S battery demonstrated two reversible cycles, while electrochemical activity of sulfur cathode was confirmed with XPS (X-ray photoelectron spectroscopy). The use of THF solvent facilitated the dissolution of sulfur and polysulfides in the electrolyte causing a discharge voltage below 1 V, and a large polarization (Li et al., 2016). A significant step forward was achieved by preparation of electrolytes from a combination of salts, specifically MgCl_2_, Mg(HMDS)_2_, and AlCl_3_ in DEG and TEG. In such electrolytes the Mg-S system displayed two discharge plateaus, the first at around 1.6 V and the second sloped plateau below 1.2 V vs. Mg/Mg^2+^. Addition of ionic liquids slightly improved the discharge capacities, but the maximum capacities did not exceed 800 mAh/g due to kinetic limitations (Zhao-Karger et al., 2014). Increase of capacities in this system was achieved by impregnation of sulfur into reduced graphene oxide, but unfortunately no data is available about the capacitance and pseudocapacitance contribution of rGO (Vinayan et al., 2016). An additional approach toward improved capacity retention is coating of separators with carbon nanofibers, which effectively suppressed the initial capacity fade from the first to the second cycle (Yu and Manthiram, 2016).

Electrolyte in Mg-S batteries plays an important role and so far different non-nucleophilic Mg electrolytes have been proposed. A large family of electrolytes is based on different combinations using Mg(TFSI)_2_ salt. Electrolytes based only on Mg(TFSI)_2_ suffer from high over potential for Mg stripping and deposition (Ha et al., 2014) due to surface passivation (Tutusaus et al., 2017). The use of a 3-electrode cell is an option for fundamental studies (Bitenc et al., 2016), whereas addition of additives that form a beneficial interlayer on the surface of Mg anode could be a solution for more realistic 2-electrode systems, as exemplified by addition of I_2_ to electrolyte (Li et al., 2017). Mg(TFSI)_2_ salt can be used to facilitate the dissolution of MgCl_2_, which is by itself insoluble in ether type of solvents (Shterenberg et al., 2015). Due to commercial availability of both salts, this is indeed one of the most common Mg electrolytes. Our group has used these two salts in combination of TEG and DOL solvents. Such combinations led to a high initial capacity of 1,320 mAh/g. The cells displayed two discharge plateaus, a high voltage plateau at 1.4 V and a lower one at 1.2 V vs. Mg/Mg^2+^ (Robba et al., 2017).

**Figure 5:**
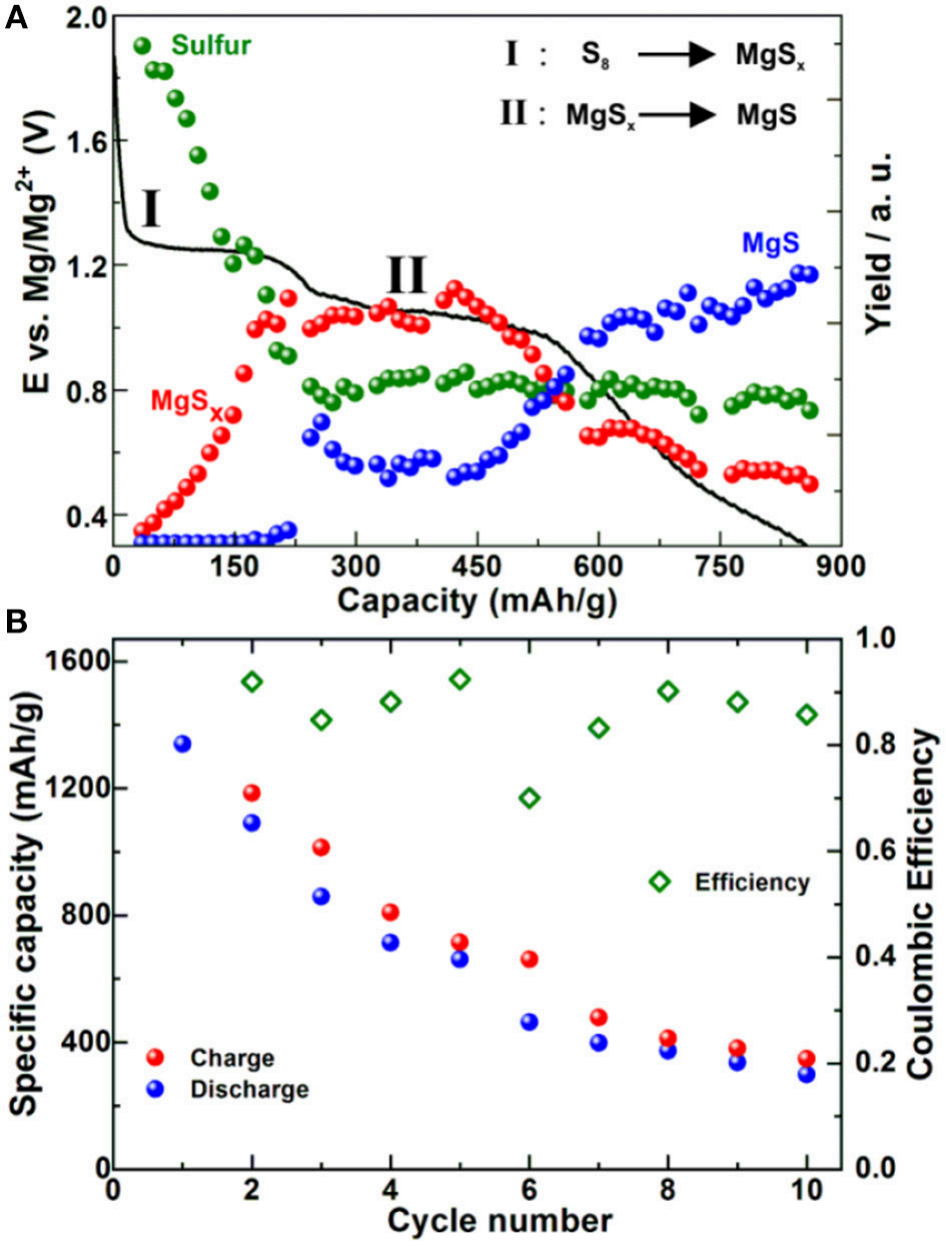
**(A)** First discharge of Mg-S battery in 0.4 M MgCl_2_-Mg(TFSI)_2_ in TEG:DOL (vol%) at C/60 and relative amounts of sulfur compounds as determined by *operando* RIXS. **(B)** Capacity and Coulombic efficiency of the same battery system tested at same electrochemical conditions. Adapted with permission from Robba et al. (2017). Copyright American Chemical Society.

By using XRD we were not able to detect any of crystalline forms of MgS, while diffraction peaks corresponding to sulfur disappeared during the first discharge plateau pointing toward electrochemical conversion of sulfur into polysulfides during the first plateau. *Operando* X-ray absorption near edge spectra (XANES) and resonant inelastic X-ray scattering (RIXS) (Figure 5A) confirmed this observation and showed that MgS precipitation starts at the beginning of the low voltage plateau. The comparison of synthesized and electrochemically prepared MgS revealed that the chemically prepared MgS crystallized in a rock-salt structure, while the electrochemically prepared analog adopted a wurtzite-like structure. The capacity fade was especially pronounced during starting cycles so that after 10 cycles the capacity value was < 400 mAh/g (Figure 5B). However, employing a highly concentrated electrolyte consisting of Mg(TFSI)_2_-2MgCl_2_ in DME improved significantly the cycling stability: more than 100 cycles with the final discharge capacity of above 600 mAh/g were achieved due to decreased solubility of polysulfides (Gao et al., 2017).

Certain reports on Mg-S batteries in tris(hexafluoroisopropyl) borate [B(HFIP)_3_] based electrolytes exhibit a peculiar single discharge plateau at approximately 1.1 V with capacities above 1,000 mAh/g and low overpotential in charge (Du et al., 2017; Xu et al., 2017; Zhang et al., 2017). It has to be noted that these systems employed Cu current collectors, which might have a crucial role in cycling, given inferior cathode performance with Al current collector (Du et al., 2017). The significant differences in this report indicate that role of Cu current collector needs to be investigated in detail and the possibility of electrochemical cycling of copper sulfide species excluded. By contrast, cycling of S cathode in similar electrolyte, Mg(B(HFIP)_4_)_2_, led to a higher voltage plateau typical for other reports on Mg–S battery (Zhao-Karger et al., 2017, 2018).

The Mg-S battery is attractive from the point of energy density and sustainability of active materials, but there are many issues which need to be resolved. For that reason, a detailed investigation of both interfacial and bulk behavior is required. It would be highly beneficial to employ more bulk-sensitive analytical techniques such as XRD, NMR, XANES, EXAFS, and RIXS, for future investigation of Mg-S electrochemistry as opposed to the currently predominating use of XPS, which gives important information about surface phenomena, but might conceal certain phenomena occurring in the bulk. Additionally, research in the field of Mg-S batteries could benefit from standardization of the electrolyte amount, which is known to have a big influence on the mechanism and performance of Li-S batteries. Since the initial report on Mg-S battery system (Kim et al., 2011) a substantial progress has been made and the specific Mg-S systems demonstrate more than 100 discharge-charge cycles (Gao et al., 2017). Regardless of that, capacity retention remains a pressing issue in most of recent reports (Li et al., 2017; Robba et al., 2017; Zhao-Karger et al., 2017). Thus, more effort should be devoted to the investigation of Mg polysulfide mechanism, as only improved understanding will provide solid guidelines toward mitigation of this issue.

## Conclusion

Several steps forward have been made in development of cathodes, especially in the performance of organic and sulfur cathodes that can be cycled for more than hundred cycles with suitable non-nucleophilic electrolytes. The progress in the field of inorganic cathodes remains more gradual with some chalcogenides surpassing the performance of Chevrel phases at elevated temperatures, while the electrochemical performance of materials based on oxide and polyanionic structures still remains limited. Although Mg battery research is almost three decades old, no standard electrolytes and testing conditions have been set so far. This is a consequence of ongoing development of new electrolytes that goes side by side with cathode research. As we have shown here, the performance of cathode materials can be largely influenced by the type of electrolyte as well as by the properties of Mg anode. Thus, researchers should compare different electrolytes and anodes but also test composites without active materials to identify possible side reactions and compatibility of electrochemical cell (housing, current collectors…). Only then standardized systems will evolve and enable a better comparison of literature results. Most importantly, the electrochemical mechanisms should be investigated through use of bulk analytical techniques to improve our understanding of electrochemical reactions inside cathodes, which will lead to development of next generation Mg cathodes.

## Author Contributions

Both authors listed have made a direct and intellectual contribution to the work, and approved it for publication.

### Conflict of Interest Statement

The authors declare that the research was conducted in the absence of any commercial or financial relationships that could be construed as a potential conflict of interest.
